# Early Patellofemoral Osteoarthritis Following ACL Reconstruction: A Narrative Review

**DOI:** 10.3390/healthcare14142081

**Published:** 2026-07-12

**Authors:** Carolina Kekki, Christoffer von Essen, Eric Hamrin Senorski, Camilo Helito, Marko Ostojic, Riccardo Cristiani

**Affiliations:** 1 Stockholm Sports Trauma Research Center, Department of Molecular Medicine and Surgery, Karolinska Institutet, 17177 Stockholm, Sweden; 2 Capio Artro Clinic, FIFA Medical Centre of Excellence, 11427 Stockholm, Sweden; 3 Department of Health and Rehabilitation, Institute of Neuroscience and Physiology, Sahlgrenska Academy, University of Gothenburg, 40530 Gothenburg, Sweden; 4 Sahlgrenska Sports Medicine Center, 40530 Gothenburg, Sweden; 5 Department of Orthopaedics, University of São Paulo, São Paulo 05508-900, Brazil; 6 Sports Traumatology Division, Traumatology Department ‘Draškovićeva’, University Hospital ‘Sisters of Mercy’, 10000 Zagreb, Croatia; 7 Osteon Orthopaedics and Sports Medicine Clinic, 88000 Mostar, Bosnia and Herzegovina

**Keywords:** patellofemoral osteoarthritis, post-traumatic osteoarthritis, anterior cruciate ligament reconstruction, risk factors, treatment

## Abstract

**Background/Objectives:** Early patellofemoral osteoarthritis (PFOA) following anterior cruciate ligament reconstruction (ACLR) is increasingly recognized as an important contributor to knee osteoarthritis (OA) in young individuals. This narrative review aimed to summarize the current evidence on early PFOA following ACLR including its prevalence, definition, risk factors, treatment, ongoing research, and future directions. **Methods:** A non-systematic literature search of PubMed/MEDLINE was conducted for English-language peer-reviewed original studies and reviews on early PFOA following ACLR, published through May 2026. Titles and abstracts were screened for relevance and full-text articles were reviewed when appropriate. ClinicalTrials.gov was searched for ongoing randomized controlled trials (RCTs). **Results:** Early PFOA following ACLR had a prevalence of 17–44% within 5 years postoperatively. Definitions remain highly heterogeneous with no universally accepted classification, although MRI-based assessment tools such as the MRI Osteoarthritis Knee Score (MOAKS) appear to be increasingly utilized. Proposed risk factors include older age, higher body mass index, concomitant meniscal injury, quadriceps weakness, altered biomechanics, and autograft choice, although findings remain inconsistent across studies. Current prevention and treatment strategies are largely extrapolated from general knee OA principles, reflecting the limited evidence specific to early PFOA. Ongoing research is increasingly focused on rehabilitation-based prevention and pharmacological interventions targeting early OA following ACLR. **Conclusions:** Early PFOA following ACLR is common in young individuals, yet evidence regarding its definition, risk factors, prevention, and treatment remains limited. The early disease stage may allow modification of structural and biomechanical changes, enabling disease-modifying pharmacological and targeted rehabilitation approaches, but standardized classification criteria and future research are needed.

## 1. Introduction

Anterior cruciate ligament (ACL) injuries are among the most common knee injuries [1]. Regardless of treatment strategy, individuals who have sustained an ACL injury have a four- to sixfold higher risk of developing long-term osteoarthritis (OA) [2]. Approximately 50% develop radiographic OA within 10–15 years following ACL reconstruction (ACLR) [1,3,4,5]. While the long-term relationship between ACL injury and OA is well-established, early OA development following ACLR has been less extensively assessed.

Most previous research has focused on non-compartment-specific knee OA or tibiofemoral OA while the patellofemoral (PF) joint has received comparatively less attention, despite being commonly affected by OA following ACLR [5,6]. In recent years, increasing attention has been directed towards early-stage OA, including patellofemoral OA (PFOA). PFOA is being increasingly recognized as an important contributor to early knee symptoms following ACLR [7,8], and previous studies have assessed early PFOA within 6 months–5 years following ACLR [9,10,11,12].

ACL injury leads to altered knee joint biomechanics, including increased anterior tibial translation [13], as well as increased lateral patellar shift and patellar tilt [14]. Although anterior tibial translation is typically restored following ACLR, other biomechanical alterations such as lateral shift and patellar tilt may remain and contribute to altered load distribution across the PF joint [14], potentially promoting the development of early PFOA. In addition, the initial injury and the associated biomechanical changes may trigger a post-traumatic biological response, including inflammatory mediator release, which can contribute to cartilage degradation and early structural changes [15].

Given that ACLR is predominantly performed in young patients, onset of OA following surgery may occur decades earlier than in the general population, potentially resulting in reduced quality of life due to pain, stiffness, functional limitations, and disability [1,3,4,5].

Emerging evidence suggests that structural changes and symptoms consistent with PFOA following ACLR may occur earlier than previously recognized [9,10,16]. Consequently, early identification of the disease is essential to implement prevention strategies and develop effective therapeutics. Early intervention in the disease process may offer a greater opportunity to modify its progression and reduce the risk of long-term consequences such as chronic pain, continued joint degeneration, and disability outcomes that are particularly impactful in the young and active population that most commonly sustain ACL injuries.

Despite this, evidence regarding the prevalence, risk factors, and management of early PFOA following ACLR remains inconsistent. In addition, there is no consensus on the definition of early PFOA, with studies using varying imaging and clinical criteria. This heterogeneity, combined with the lack of a structured synthesis of the available evidence, limits clinical interpretation and hinders the development of targeted prevention and management strategies.

In this narrative review, early PFOA was defined by structural changes including cartilage lesions, bone marrow lesions, osteophytes, and joint space narrowing on either plain radiography, MRI, or second-look arthroscopy, assessed using standardized grading systems. Clinical early PFOA was defined based on studies applying either the original Luyten criteria [17] or the updated Luyten criteria [18]. Studies with assessment within 5 years of ACLR were included.

The aim of this narrative review was to synthesize the current evidence on the prevalence, definition, risk factors, and management of early knee PFOA following ACLR, as well as to describe ongoing research and highlight important directions for future research.

## 2. Materials and Methods

A non-systematic literature search was conducted in PubMed/MEDLINE to identify relevant studies on early PFOA following ACLR published up to May 2026. The search combined Medical Subject Headings (MeSH) terms and free-text keywords related to “anterior cruciate ligament reconstruction”, “ACLR”, “patellofemoral osteoarthritis”, “early patellofemoral osteoarthritis”, “PFOA”, “knee osteoarthritis”, “post-traumatic osteoarthritis”, and “early post-traumatic osteoarthritis”, as well as terms related to epidemiology, risk factors, biomechanics, diagnosis, prevention, and treatment. A representative search strategy included combinations of terms such as: (“anterior cruciate ligament reconstruction” OR “ACLR”) AND (“patellofemoral osteoarthritis” OR “early patellofemoral osteoarthritis” OR “PFOA” OR “post-traumatic osteoarthritis”) AND (risk factors OR biomechanics OR prevention OR treatment).

Additionally, ClinicalTrials.gov was searched to identify ongoing randomized controlled trials (RCTs) investigating OA prevention following ACLR. The condition/disease searched was “osteoarthritis, knee”, with the additional term “anterior cruciate ligament reconstruction”. Studies focusing on early degenerative changes or OA were considered.

English-language peer-reviewed original studies and systematic reviews were considered. Study protocols from ongoing studies found on ClinicalTrials.gov were also considered. Eligible studies included patients who had undergone ACLR, with outcomes related to early PFOA, structural PF joint changes, or clinically defined early OA. Studies focusing exclusively on tibiofemoral OA without PF assessment were excluded. Early PFOA was defined by structural changes including cartilage lesions, bone marrow lesions, osteophytes, and joint space narrowing on either plain radiography, MRI, or second-look arthroscopy, assessed using standardized grading systems. Clinical early PFOA was defined based on studies applying either the original Luyten criteria [17] or the updated Luyten criteria [18]. Studies with assessment within 5 years of ACLR were included. No restrictions were placed on study design; however, emphasis was placed on cohort studies, case–control studies, RCTs, and high-quality systematic reviews. Studies including non-operated ACL injuries were included only if PF outcomes were reported separately for ACLR cohorts.

Titles and abstracts were screened for relevance, followed by full-text evaluation where appropriate. Study selection was performed iteratively, with additional relevant articles identified through reference list screening of included studies.

Due to the narrative nature of this review, no PRISMA flow diagram was generated. However, risk of bias was assessed for all studies included in the risk factor section of the Results using the Cochrane Risk Of Bias In Non-randomized Studies of Interventions I version 2 (ROBINS-I V2) [19], and can be found in the electronic Supplementary Material Figure S1. The assessment included six domains: bias due to confounding, classification of interventions, selection of participants, missing data, measurement of outcomes, and selection of the reported result. Each domain was rated as possessing low, moderate, serious, or critical risk of bias.

The final synthesis was structured thematically into definitions, diagnosis, prevalence, risk factors, prevention, treatment, and ongoing research.

## 3. Results

### 3.1. Definition

The definition and diagnosis of early PFOA following ACLR remain heterogenous and poorly standardized. The concept of early PFOA must be clarified, as it may refer either to early onset after surgery, or to an early stage in the disease progress. In this narrative review, early PFOA was defined by structural changes including cartilage lesions, bone marrow lesions, osteophytes, and joint space narrowing on either plain radiography, MRI, or second-look arthroscopy, assessed using standardized grading systems. Clinical early PFOA was defined based on studies applying either the original Luyten criteria [17] or the updated Luyten criteria [18]. Only studies with assessment within 5 years of ACLR were included. Previous studies have assessed early PFOA within 6 months–5 years following ACLR [9,10,11,12]. Although a 5-year follow-up is often considered mid-term in ACLR research, this timeframe is appropriate given the slow progression of osteoarthritis and is consistent with definitions used in studies of early PFOA [9,10,11,12].

Conventional diagnostic criteria for knee OA, including the Kellgren–Lawrence classification [20] and the American College of Rheumatology criteria [21], are widely used to identify established disease but have important limitations in early PFOA. In particular, the Kellgren–Lawrence classification is validated for tibiofemoral OA [22], but lacks sensitivity for early structural changes in the PF joint. Similarly, the Iwano classification [23] is specific to the PF joint but is based solely on radiographic joint space narrowing, limiting its utility for early disease detection. There are also other, less-utilized radiographic criteria such as the modified Outerbridge criteria [24] focusing on cartilage loss.

### 3.2. Diagnosis

#### 3.2.1. Clinical Diagnosis

Several clinical criteria for early knee OA have been proposed. One of the earliest was introduced by Luyten et al. in 2012 [17], combining knee pain with a Kellgren–Lawrence grade ≤ 2 and arthroscopic or magnetic resonance imaging (MRI) evidence of cartilage degeneration and/or subchondral bone marrow lesions. Updated Luyten criteria in 2018 [18] placed greater emphasis on clinical features, requiring a Knee injury and Osteoarthritis Outcome Score (KOOS) subscale score ≤ 85 on at least two of four subscales (excluding Sport and Recreation), together with joint line tenderness or crepitus, and a Kellgren–Lawrence grade ≤ 1. Mahmoudian et al. [25] expanded these criteria by additionally including joint effusion and Heberden’s nodes. In 2017, the Italian Society for Rheumatology [26] proposed an alternative definition based on knee pain and/or stiffness and risk factors. More recently, the Osteoarthritis Research Society International (OARSI) identified 77 candidate items for early knee OA through a Delphi process [27], illustrating the heterogeneity of early disease presentation and the challenge of establishing standardized criteria. Existing clinical criteria combine symptoms, clinical findings, radiographic features, and patient-reported outcomes, but are not specific to the PF joint and remain variable across studies.

#### 3.2.2. Radiologic Diagnosis

As interest in early knee OA has increased, the use of MRI-based scoring systems have been increasingly used to define early structural changes [28]. The most commonly used MRI-based classification for early knee OA is the MRI Osteoarthritis Knee Score (MOAKS) [29], used in several previous studies [30,31,32]. The MOAKS divides the knee into 14 subregions, including four PF compartments (medial/lateral patella and medial/lateral trochlea). It evaluates articular cartilage, bone marrow lesions, cysts, osteophytes, meniscal pathology, synovitis/effusion, and ligaments/tendons, and periarticular features including pes anserine bursitis, iliotibial band signal, popliteal cysts, infrapatellar bursa signal, prepatellar bursa signal, and ganglion cysts. Each subregion is rated on a scale from 0 (normal/no pathology) to 3 (severe pathology), providing a whole-joint assessment. MOAKS allows detection of subtle knee joint changes relevant to early PFOA, and is based on the previous classification systems Whole-Organ Magnetic Resonance Imaging Score (WORMS) [33] and Boston Leeds Osteoarthritis Knee Score (BLOKS) [34]. The MOAKS is more suitable for evaluating early knee joint changes than WORMS and BLOKS as it was developed to address limitations in these classification systems through improved subregional scoring, reduced redundancy, and refined assessment of bone marrow lesions [29]. However, the use of MRI is limited by availability constraints, high costs, and variability in image interpretation between observers. In contrast, radiographic classification systems such the modified Outerbridge criteria [24] and the Kellgren–Lawrence [20] and Iwano classifications [23] are more widely available but cannot assess soft tissue pathology or cartilage status, and are therefore less sensitive than MRI for detecting early PFOA. However, the Iwano classification may still be useful in clinical criteria by excluding established PFOA.

Lastly, a less commonly used method for assessing early PFOA is second-look arthroscopy, which enables direct evaluation of cartilage lesions. However, because it is an invasive procedure, its use is limited, and it is not suitable for routine assessment of all patients. Clinical and radiologic criteria for early PFOA vary considerably, reflecting heterogeneity across current classification systems. This limits comparability across studies and hampers consistent definition of study populations in research on early PFOA, underscoring the need for standardized diagnostic criteria for early PFOA following ACLR.

### 3.3. Prevalence of PFOA

Early PFOA following ACLR is common, although the reported prevalence varies widely depending on definition and diagnostic method. At 1 year postoperatively, prevalence ranges from 17% [9,35] to 30% [32], with estimates of 38% at 2 years [30] and 21% at 3 years [36]. One study reported worsening PF cartilage defects in 44% of patients between 1 and 5 years following ACLR based on MOAKS [11]. For symptomatic early OA, two studies utilizing the Luyten 2018 criteria [18] reported prevalences of 54% at 6 months [12] and 22% at both 6 and 12 months [16] following ACLR, while another recent study reported 68% at 6 months, 54% at 1 year, and 46% at 2 years [10]. However, this study defined early OA as ≤85% on at least two of four KOOS subscales and is thus not specific to PFOA per se. A summary of the studies assessing the prevalence of early PFOA following ACLR is presented in Table 1. The variability in reported prevalence may be explained by several factors. Symptom-based classifications assess the whole knee rather than PFOA specifically, which may partly explain the higher prevalence in studies using clinical criteria. Differences in diagnostic criteria, imaging modalities, definitions of early PFOA, and differences across studies in patient populations and surgical techniques further contribute to heterogeneity.

### 3.4. Risk Factors

Several previous studies have investigated potential risk factors for early PFOA following ACLR. These include both non-modifiable and modifiable factors, spanning patient characteristics, injury-related variables, surgical factors, and postoperative functional and biomechanical outcomes, with findings remaining inconsistently reported across studies. The following sections summarize the current evidence for the most commonly studied risk factors for early PFOA. A summary of all studies included in this section can be found in the Supplementary Material, Table S1.

The available evidence suggests that older age at the time of ACLR may be associated with an increased risk of early PFOA. One study reported that older age was significantly associated with increased odds of PF cartilage lesions at 1 year following ACLR [9], while another reported significantly increased odds of PFOA at 2 years [36] postoperatively by second-look arthroscopy. Patterson et al. [11] further reported that age > 26 years at ACLR was associated with greater odds of PF cartilage worsening at 1–5 years postoperatively. As increasing age is one of the most prominent risk factors for established knee OA [37], these findings are not surprising. Older patients may have greater pre-existing PF joint degeneration and reduced biological repair capacity, making the joint less resilient to cartilage thinning, muscle weakness, and oxidative damage, all of which may contribute to the development of early PFOA.

The role of sex remains unclear, with conflicting evidence regarding its association with early PFOA. One study reported male sex to be associated with a five- to sixfold increased odds of PFOA at 1 year following ACLR [9], another reported that 79% of patients with early PFOA at 1 year postoperatively were male [32], and a third identified male sex as a significant risk factor for PF cartilage lesions at 18 months postoperatively [38]. In contrast, other studies have reported no association between sex and the risk of early PFOA following ACLR [30,36], despite primary isolated PFOA (without previous ACL injury or ACLR) being more prevalent among females [39,40].

Overweight and obesity are well-known risk factors for established knee OA [37], and two studies on early PFOA following ACLR are consistent with this association [9,41]. Increased Body Mass Index (BMI) contributes to established knee OA through higher PF joint forces and mechanical stress [42], and may also promote knee OA through systemic metabolic effects, including elevated levels of pro-inflammatory mediators associated with excess adiposity [43]. However, there are also studies that have found no significant association between BMI and early PFOA following ACLR [30,36], and there is a need for further research on early PFOA assessing BMI. Although specific associations and mechanisms linking higher BMI to early PFOA development are not yet fully evaluated and understood, they are likely similar to those implicated in established knee OA [44,45,46].

Concomitant meniscal injuries are common upon primary ACLR [47,48] and are well-established contributors to both long-term knee OA and PFOA following ACLR [2,49,50]. However, their role in early PFOA remains less clear. Li et al. [30] identified partial meniscectomy as a predictor of early PFOA, while Huang et al. [51] reported partial lateral and medial meniscal resection upon primary ACLR to be significantly associated with patellar cartilage damage at one year following ACLR. Another study reported that concurrent meniscectomy, but not meniscal repair, upon primary ACLR was a significant predictor of PFOA at 3 years follow-up [36], while a fourth study reported no association between PF cartilage damage and either meniscal injury or treatment at 1 year postoperatively [38]. Overall, evidence regarding the impact of concomitant meniscal injury and its management on early PFOA remains limited and inconsistent.

A greater time from injury to ACLR has been associated with an increased risk of secondary knee injuries [48,52] and long-term OA [53]. However, evidence regarding early PFOA is inconsistent. One study reported delayed ACLR to be associated with early PFOA [30], whereas an RCT reported greater PF cartilage thickness loss following early ACLR across both 2 and 5 years postoperatively [54]. Other studies found no association between surgical timing and early PFOA [9,55]. Thus, the influence of ACLR timing on early PFOA remains unclear, highlighting the need for further studies to clarify this relationship.

Few studies have evaluated the impact of autograft choice on early PFOA following ACLR. Long-term studies suggest that bone–patellar tendon–bone (BPTB) autografts may be associated with PF joint degeneration [56,57], potentially due to donor-site morbidity and altered PF biomechanics following autograft harvest. A randomized study by Frobell et al. [58] remains one of few studies evaluating the association between autograft type and PFOA following ACLR. It reported higher prevalence of radiographic PFOA following ACLR with BPTB compared with hamstring tendon (HT) autografts at 5 years follow-up, although it did not evaulate early PFOA per se. A more recent study reported no association between autograft choice (BPTB or HT) and PF cartilage changes at 1 year postoperatively [38]. To the authors’ knowledge, no studies have specifically evaluated the association between QT autografts and early PFOA following ACLR. The limited evidence and increased use of QT autografts [59] highlights the need for future research.

Quadriceps weakness is common following ACLR [60,61] and has been proposed as a risk factor for early PFOA. Isokinetic quadriceps weakness has been reported to predict early PFOA [36], and has been associated with worsening PF cartilage status at 1 year following ACLR [38]. Reduced quadriceps strength, defined as <80% of the contralateral limb, has also been associated with worsening PF cartilage damage at 2 years postoperatively [51,62]. Two further studies reported significant associations between early PFOA and limb symmetry index [63] or thigh circumference [30], the latter serving as a surrogate measure of quadriceps strength. Overall, the available evidence consistently suggests that quadriceps weakness is associated with early PFOA or progression of PF cartilage damage, although outcome measures vary considerably across studies.

While ACLR restores anterior and rotatory knee laxity associated with ACL injury [14], other biomechanical abnormalities may persist and contribute to early PFOA. Van de Velde et al. [14] reported persistent patellar rotation, patellar tilt, and lateral shift in cartilage contact at 6 months after ACLR. Subsequent studies have similarly associated patellar rotation [64,65], lateral patellar tilt [65,66], and lateral displacement [35,66] with early structural changes in the PF joint. Several studies have also evaluated PF joint contact forces during level walking [8], running [67,68,69], and single-leg hop tests [32,64,70]. One study reported PF joint contact forces in the ACL reconstructed knee to be smaller during the first 6 months compared to the uninjured knee and controls, but comparable at 24 months [8], whereas others reported lower PF joint contact forces in the operated knee at 12–24 months [69,70] and 4.5 years [67]. Lower peak PF joint contact forces during a single-leg hop test have been associated with both early PFOA at 1 year and worsening PFOA between 1 and 5 years postoperatively [32]. Another study reported smaller peak knee flexion angles and greater knee internal rotation excursion in patients with early PFOA during a forward hop task at 1–2 years following ACLR, compared with patients without early PFOA [64]. Despite substantial heterogeneity in biomechanical measures and assessment methods, the available evidence suggests that altered biomechanics following ACLR may contribute to early PFOA.

Overall, the available evidence is most consistent for quadriceps weakness and altered biomechanics as risk factors for early PFOA following ACLR, with multiple studies reporting associations despite heterogeneity in assessment methods and outcome measures. Older age and BMI may also be associated with increased risk; however, the supporting evidence is more limited. In contrast, findings regarding sex, concomitant meniscal injury, timing of ACLR, and autograft choice remains inconsistent, with conflicting results across studies. Collectively, these findings suggest that modifiable functional factors, particularly quadriceps weakness and biomechanical alterations, currently have the most consistent evidence base, whereas demographic, surgical, and injury-related factors remain less certain.

### 3.5. Prevention

Secondary prevention of early PFOA refers to efforts to slow or halt the progression of symptoms and structural changes in individuals with early manifestations or at elevated risk. However, evidence specifically evaluating prevention and treatment strategies for early PFOA following ACLR remains very limited. Therefore, to provide clinically relevant recommendations, evidence from contemporary early knee OA guidelines was included, as this currently represents the best available evidence to guide management of early PFOA following ACLR. In recent years, two major consensus guidelines on secondary prevention of early OA have been published: the OPTIKNEE consensus [71] and the OA Action Alliance (OAAA) recommendations [72,73].

OPTIKNEE provides recommendations for patients with traumatic knee injuries, including but not exclusive to ACL injuries, with emphasis on those who remain symptomatic beyond usual recovery. It was developed using a hybrid consensus model, in which five guiding questions were developed to achieve consensus objectives. Systematic reviews were conducted to synthesize evidence for each question before consensus definitions and recommendations were finalized. The guideline includes eight clinical and six research recommendations, focusing primarily on symptomatic post-traumatic OA rather than radiologic signs based on the rationale that pain, disability, and impaired quality of life drive OA burden, whereas the relationship between symptoms and radiologic changes is inconsistent. The clinical recommendations address patient selection, timing of interventions, and outcome monitoring to reduce the burden of symptomatic OA.

The OAAA similarly provides consensus-based recommendations, but focuses specifically on secondary OA prevention in patients ≤40 years following ACL injury. It includes 15 clinical recommendations addressing patient education, exercise and rehabilitation, psychological skills training, graded-exposure therapy, outcomes to monitor, secondary injury prevention, system-level social support, leveraging technology, and coordinated care models. The aim is to prevent or delay symptomatic OA onset, reduce disability, and improve quality of life.

Across both frameworks, exercise-based rehabilitation is the central intervention strategy. In clinical practice, this translates into progressive strengthening and neuromuscular rehabilitation targeting restoration of quadriceps and hamstring function, knee range of motion, and movement quality. These programs typically progress from supervised rehabilitation to independent exercise and incorporate weight-bearing strengthening, neuromuscular control training, and plyometric and sport-specific loading. Return to pivot sports should be delayed until at least 9 months after surgery, and should be based on successful completion of a comprehensive return-to-sport test battery rather than time alone.

Patient education and self-management are considered essential components of secondary prevention. This includes education regarding injury-related OA risk, load management, symptom monitoring (e.g., effusion and pain flares), and long-term modifiable risk factors such as quadriceps weakness, high-impact loading, and physical inactivity. These elements are intended to support adherence and long-term behavioral change rather than short-term rehabilitation alone.

The OPTIKNEE and OAAA represent the most comprehensive consensus efforts to date in secondary prevention of early knee OA, although both are constrained by an underlying paucity of high-quality evidence. Moreover, neither guideline specifically addresses early PFOA.

### 3.6. Treatment

Evidence specifically addressing treatment strategies for early PFOA following ACLR is limited. Most treatments are general for knee OA overall rather than specific to PFOA, although some have been specifically evaluated for PFOA, such as bracing and orthotics [74,75], as well as some intra-articular injections [76,77]. Therefore, evidence from contemporary knee OA studies and guidelines was also included, as this currently represents the best available evidence to guide management of early PFOA following ACLR. Surgery is not a first-line option for early PFOA, but can be considered in patients with established PFOA and persisting symptoms despite non-surgical treatment [78,79]. Hence, surgical treatment will not be further discussed in this section.

#### 3.6.1. Non-Operative Management

Non-operative management of early PFOA is mainly extrapolated from general knee OA treatment principles, and typically includes patient education, weight management, and structured physiotherapy, with particular emphasis on quadriceps strengthening [78,79,80], as mentioned in the Prevention section above. Knee braces and orthotics are among the few interventions specifically evaluated in PFOA, and aim to reduce pain and modify patellar alignment to reduce joint contact forces on the lateral PF compartment, which is most commonly affected. However, the evidence supporting their use is scarce and inconsistent [74,75], and available studies have primarily included patients with established rather than early PFOA. Accordingly, clinical guidelines provide only conditional [80] or moderate [79] recommendations for PF bracing and orthotics in PFOA management.

#### 3.6.2. Pharmacological Management

NSAIDs, topical and/or oral, are strongly recommended as first-line pharmacological treatment for knee OA due to their analgesic and anti-inflammatory properties [78,79,80], but should be used at the lowest effective dose for the shortest duration possible due to increased risk of cardiovascular thrombotic and gastrointestinal events [79]. Paracetamol is also commonly used and may improve pain and function without increased risk of adverse events [79,81], although it is not strongly recommended in the OARSI 2019 guidelines due to limited efficacy [78]. Duloxetine, a serotonin–norepinephrine reuptake inhibitor, may be used in patients with persistent pain or concomitant central pain sensitization as it improves pain and function in knee OA [78,80,82], but is not considered first-line due to tolerability issues and adverse effects including nausea and gastrointestinal symptoms [82]. Two Cochrane reviews on Tramadol [83] and opioids [84] reported no to small benefits in knee OA, with increased risks of adverse events including nausea, vomiting, lightheadedness, dizziness, headache, and potential addiction. Thus, neither Tramadol nor opioids are recommended as standard treatment [78,79,80], although they may be considered when other pharmacological options fail and surgery is not feasible [80].

Intra-articular injections of various agents represent another treatment option for knee OA. Corticosteroids are the most commonly used and are recommended for short-term pain relief [78,79,80]. Hyaluronic acid injections may improve pain and function in PFOA [76,77], and were recommended by the 2019 OARSI guidelines [78], although the American Academy of Orthopaedic Surgeons (AAOS) [79] and American College of Rheumatology (ACR) [80] guidelines had a moderate recommendation against their use due to limited evidence of efficacy. Platelet-rich plasma (PRP) is another intra-articular option and is hypothesized to promote chondrogenesis, cell proliferation, angiogenesis, cell differentiation, and bone remodeling in knee OA. However, the supporting evidence is of limited quality [85,86], and the OARSI [78] and ACR [80] guidelines strongly recommended against its use due to heterogeneity and lack of standardization in preparation and technique.

### 3.7. Ongoing Research

#### 3.7.1. Prevention

There are several ongoing RCTs investigating prevention of early OA following ACLR through rehabilitation strategies, although none specifically address early PFOA.

The Stop OsteoARthritis (SOAR) study [87] evaluates a 6-month digital education and exercise program aimed at improving quadriceps strength in individuals at increased risk of early OA, compared with minimal intervention. Outcomes are assessed using patient-reported outcomes at 6 months and MRI at 24 months using MOAKS. The SUpervised exercise-therapy and Patient Education Rehabilitation (SUPER)-Knee trial [88] compares a structured exercise and education intervention with minimal care in patients with persistent symptoms (KOOS_4_ < 80) at 9–36 months following ACLR. Two additional RCTs aim to modify knee biomechanics and joint loading following ACLR. One evaluates an 8-week active feedback gait retraining program, measuring knee joint loading at 8 weeks and 6 months, including MRI evaluation at 6 months [89]. The second evaluates a 12-week personalized sensor-based exercise intervention targeting pathological movement patterns, assessing knee joint load at 6 months and MRI outcomes including MOAKS at 6 and 36 months [90]. Finally, an observational prospective cohort study aims to develop computational models to predict cartilage degeneration and pain progression in patients with an ACL injury with or without ACLR, and in patients with early-stage knee OA [91]. This study integrates data from MRI, movement analysis, muscle strength testing, patient-reported outcomes, and synovial and plasma biomarkers with follow-up assessments at baseline, 1, and 3 years, with extended follow-up planned at 7 and 10 years.

Collectively, these studies have potential to provide important insights into prevention of early knee OA following ACLR, as several studies include measures capable of assessing different compartments of the knee. They also align with prior recommendations emphasizing the need for early intervention strategies [71].

#### 3.7.2. Pharmacological Management

There are yet no approved drugs for early OA that reverse, halt, or slow down disease progression, and previous clinical trials attempting to develop such drugs have largely been unsuccessful [92]. One possible explanation is that most prior studies have included patients with established knee OA, where joint damage may already be irreversible. The definition and use of early OA or PFOA may therefore offer an opportunity for earlier intervention, when structural damage is less advanced and disease progression may be more modifiable.

Several ongoing RCTs are evaluating pharmacological strategies in early knee OA. These include intra-articular interventions such as PRP [93,94], bone marrow concentrate [94], and interleukin-based therapies [95]. Other RCTs evaluate the effect of oral pharmacological agents, including Tranexamic acid [96], Montelucast [97], and Metformin [98]. A common feature across these RCTs is their explicit focus on early or post-traumatic knee OA, with follow-up periods ranging from 6 months to 2 years. This reflects a growing interest in early knee OA as a target for disease-modifying interventions.

Notably, none of these RCTs specifically target early PFOA, although several include MRI evaluations able to capture early structural changes in different knee compartments. Whether these studies will ultimately lead to clinically available disease-modifying pharmacological treatment for early knee OA or PFOA remains uncertain. Nevertheless, the increasing number of pharmacological RCTs on this topic is encouraging and reflects the growing interest in early knee OA.

## 4. Discussion

### 4.1. Definition and Diagnosis

Although early PFOA is increasingly recognized as an important component of knee OA with a higher prevalence than previously assumed [5,6], its definition and diagnosis remain poorly standardized. Unlike the Kellgren–Lawrence system [20], which is neither validated for or specific to the PF joint, the Iwano classification [23] was developed for PFOA but is infrequently used in studies of early disease. Instead, Kellgren–Lawrence grades ≤1–2 are commonly applied to exclude established OA, although this may not adequately capture changes specific to the PF joint.

Substantial heterogeneity in diagnostic criteria across studies of early PFOA following ACLR further limits comparability across studies. This reflects the lack of a unified framework, with studies differing in their reliance on radiographic, MRI-based, or symptom-based definitions. In primary care settings, there is likely a need for accessible, radiograph-based criteria, potentially including the Iwano classification alongside relevant clinical symptoms. In contrast, research settings require higher specificity, with MRI-based approaches such as MOAKS [29] commonly used in previous and ongoing studies [30,31,32,87,89,90]. Furthermore, better characterization of symptoms associated with early PFOA is needed to support future diagnostic and classification criteria. The OARSI initiative recently identified candidate items for early OA, including 23 symptom-related items [27], and a similar approach may be useful for early PFOA. Overall, developing and validating universally accepted classification criteria that integrate imaging and clinical features would improve case identification and facilitate comparisons across studies.

### 4.2. Risk Factors

Early PFOA following ACLR appears to have a multifactorial etiology involving non-modifiable and modifiable risk factors. This section focuses on the modifiable risk factors. Elevated BMI is a well-established risk factor for knee OA and may contribute to early PFOA through similar mechanisms. However, given its well-established role in knee OA and limited specificity to early PFOA, the discussion focuses on other modifiable factors more directly related to early PFOA.

Concomitant meniscal injury is a recognized contributor to knee OA and PFOA [2,49,50], and a longer interval to ACLR has been associated with an increased risk of secondary meniscal damage [48,52], further increasing the risk of degenerative changes. Meniscectomy may contribute to PFOA through altered knee biomechanics and increased PF joint loading postoperatively [99], and has been suggested to influence PFOA independently of tibiofemoral OA changes [100]. Although evidence specific to early PFOA remains limited, these findings suggest that early ACLR and meniscus preservation when feasible may be relevant in reducing the risk of early PFOA.

Autograft choice represents another intraoperative, modifiable factor that remains insufficiently studied in relation to early PFOA. Some studies have suggested an increased risk of PFOA with BPTB autografts [56,57], but comparisons were made only with HT autografts. The QT autograft has gained increasing popularity in recent years [59], while evidence regarding its association with early PFOA remains limited. Further studies are needed to clarify the impact of autograft choice on early PFOA following ACLR, as this represents a potentially modifiable surgical factor with clinical relevance.

The QT autograft may be of particular interest in this context, as quadriceps weakness has been proposed as a risk factor for early PFOA in several studies [36,38,51,62]. Supporting this, one study reported greater quadriceps weakness at 7 months following ACLR with a QT autograft compared with HT and BPTB autografts [101]. Whether these deficits persist over time remains unclear and warrants further investigation. Existing studies examining the association between quadriceps strength and early PFOA are relatively small, with sample sizes ranging from 78 [63] to 177 [30] participants. Given that postoperative strength testing, including isokinetic assessment, is performed in clinical return-to-sport protocols, there is potential to establish larger longitudinal cohorts to further clarify the association between quadriceps weakness and early PFOA. Building on this need for higher-quality evidence, an ongoing RCT is evaluating whether a 6-month digital education and exercise program targeting quadriceps strengthening can reduce the risk of early PFOA following ACLR [87]. Such studies align with a secondary prevention perspective and may improve understanding of modifiable risk factors for early PFOA.

During weight acceptance, the limb accepts full support of the body and attenuates shock through knee flexion that is controlled by an eccentric contraction of the quadriceps.

Quadriceps weakness may also contribute to altered biomechanics following ACLR. Smaller peak knee flexion angles have been associated with quadriceps weakness following ACLR [102], as the weight acceptance during gait is primarily controlled through knee flexion, which is facilitated by an eccentric contraction of the quadriceps. Therefore, greater quadriceps strength may facilitate attenuation of landing forces through a softer landing strategy involving greater knee flexion angles. Reduced knee flexion limits the knee’s ability to absorb landing forces [103], thereby increasing reliance on passive structures and potentially elevating joint stress. Given that quadriceps weakness partly drives these movement patterns, it may represent an important modifiable factor contributing to altered joint loading and the risk of early degenerative changes following ACLR. Quadriceps weakness may also contribute to the underloading reported by studies assessing PF joint contact forces during walking [8], running [67,68,69], and single-leg hop tests [32,64,70], which may be clinically relevant for cartilage degeneration considering the load-dependent nature of articular cartilage. Furthermore, persistent alterations in patellar rotation [64,65], lateral patellar tilt [65,66], and lateral displacement [35,66] may lead to abnormal PF joint loading and contribute to early degenerative changes. Increased patellar rotation may alter patellar tendon orientation relative to the tibia long axis [14], thereby influencing PF joint load. Similarly, increased lateral patellar tilt and lateral displacement may increase loads on the lateral aspect of the knee and contribute to maltracking of the patella, potentially affecting the PF joint load distribution [65,66]. Ongoing RCTs are evaluating gait retraining [89] and sensor-based movement correction programs [90], which may help address persistent biomechanical alterations through more individualized rehabilitation approaches, but a more comprehensive understanding of the association between biomechanical alterations, quadriceps weakness, and early PFOA is needed.

### 4.3. Prevention

Despite the existence of comprehensive consensus guidelines for secondary prevention of knee OA following ACL injury and ACLR [71,72,73], these guidelines are not specific to PFOA. This represents an important gap, as the PF joint appears particularly susceptible to early degenerative changes after ACLR, yet it is not explicitly targeted in current prevention strategies.

Ongoing RCTs evaluating structured exercise-based rehabilitation programs [87,88], gait retraining [89], and sensor-based movement correction programs [90] may contribute important insights into the prevention of early PFOA following ACLR. Although these studies are not explicitly designed to evaluate PFOA, they incorporate MRI-based assessments, enabling evaluation of early structural changes within the PF joint. If effective, these interventions may inform both general rehabilitation strategies and more targeted approaches aimed at modifying biomechanical risk factors associated with early PFOA.

In addition, one ongoing study aims to use computational models to predict cartilage degeneration by integrating MRI, movement analysis, muscle strength, patient-reported outcomes, and synovial and plasma biomarkers. This represents a step toward more individualized risk stratification following ACL injury and ACLR. Such approaches may ultimately enable earlier identification of high-risk individuals and support more tailored rehabilitation strategies aimed at preventing post-traumatic joint degeneration, including early PFOA. More broadly, predictive modeling and patient phenotyping may represent an important future direction in both PF and general knee osteoarthritis prevention.

### 4.4. Treatment

Evidence specific to treatment for early PFOA following ACLR remains scarce. Current management strategies are largely extrapolated from general knee OA. Bracing and orthotics are among the few interventions evaluated for early PFOA and may influence joint loading patterns, although current guidelines only provide conditional [80] or moderate [79] recommendations for their use. Regarding pharmacological treatment, one small pilot study including 43 patients evaluated intra-articular hyaluronic acid injections for PFOA and reported significant improvement at 4–26 weeks [77]; however, the authors highlighted the need for adequately powered RCTs to further assess its efficacy.

Studies focusing specifically on early PFOA remain scarce, despite the possibility that structural changes may be most modifiable at this stage of disease. Several ongoing RCTs are evaluating pharmacological interventions targeting early or post-traumatic knee OA, including both oral medications [57,96,97] and intra-articular injections [93,94,95]. These interventions aim to reduce cartilage degeneration following ACLR or modulate the inflammatory response implicated in early OA development. Research targeting early PFOA, and early OA in general, represents an important step toward the development of future potentially disease-modifying treatments for knee OA.

### 4.5. Strengths

This narrative review summarizes the available evidence on early PFOA following ACLR, an emerging but insufficiently studied topic. To the authors’ knowledge, this is the first comprehensive synthesis focusing specifically on early PFOA following ACLR, integrating evidence from epidemiological, imaging, biomechanical, and clinical studies. A strength of this review is the structured approach used to define early PFOA, incorporating both structural and clinical criteria, which improves comparability across heterogeneous studies. In addition, the review synthesizes evidence across multiple domains, including prevalence, risk factors, biomechanics, and management, providing a clinically oriented overview of the literature. Finally, this review highlights key gaps in the current evidence base and outlines directions for future research.

### 4.6. Limitations

This narrative review has several limitations. First, as a narrative rather than a systematic review, the search strategy and study selection were not fully exhaustive or protocol-driven, introducing a potential risk of selection bias. Second, there is a paucity of studies specifically assessing early PFOA following ACLR, which limits the strength of conclusions that can be drawn. In addition, the included studies were heterogeneous in terms of study design, patient populations, definitions of early PFOA, and outcome measures, limiting comparability and synthesis across studies. Third, the majority of included studies were observational, with relatively few RCTs, limiting causal inference regarding risk factors and management strategies. Furthermore, variability in imaging modalities, diagnostic criteria for early PFOA, and follow-up duration after ACLR may have influenced reported prevalence estimates and observed associations. Finally, despite efforts to comprehensively synthesize the available literature, relevant studies may have been missed due to the non-systematic search approach.

## 5. Conclusions

Early PFOA following ACLR is common and represents one of the most debilitating subgroups of knee OA, affecting a young and often physically active population and leading to pain, stiffness, functional limitations, and reduced quality of life early in life. At the same time, early PFOA presents a unique opportunity to advance understanding of PFOA pathogenesis, risk factors, and potential treatment strategies. The early stage of disease may provide a window during which structural and biomechanical changes remain modifiable. This creates potential for the development of disease-modifying pharmacological interventions and targeted rehabilitation strategies aimed at slowing or preventing disease progression. However, current evidence remains limited regarding definition, prevention, and treatment of early PFOA, although several ongoing RCTs are investigating preventive and therapeutic interventions targeting early disease stages.

## 6. Future Directions

Future research on early PFOA following ACLR should focus not only on treatment strategies, but also on improving the fundamental understanding and definition of the condition. The substantial heterogeneity in diagnostic criteria across current studies highlights the need for universally accepted classification systems integrating imaging findings with clinically relevant symptoms, adapted to both primary care and specialized clinical settings. Such criteria would improve comparability across studies and facilitate recruitment of more homogenous research populations.

In parallel, larger longitudinal studies are needed to clarify the contribution of modifiable risk factors, including autograft choice, quadriceps weakness, altered biomechanics, and concomitant meniscal injury, to the development of early PFOA. Emerging approaches, including quantitative MRI, biomechanical modeling, and predictive frameworks integrating imaging, movement analysis, muscle strength, and patient-reported outcomes, may further improve identification of individuals at high risk of early PFOA and facilitate more personalized prevention and rehabilitation strategies.

Finally, ongoing RCTs evaluating secondary preventive strategies, rehabilitation strategies, alterations of biomechanics, and pharmacological treatments represent an important step toward more targeted management of early PFOA. As structural changes may be more modifiable in early OA, before irreversible joint damage occurs, improved definition of early PFOA may facilitate the development of preventive and potentially disease-modifying treatment strategies in the future.

## Figures and Tables

**Table 1:** Prevalence of early PFOA following ACLR.

Study	Year	Study Design	Diagnostic Method PFOA	Number of Patients (% Male)	Mean Age	Follow-Up	Prevalence
Culvenor et al. [9]	2015	Cross-sectional	MOAKS	111 (64%)	30 ± 8	1 year	17%
Schache et al. [32]	2023	Prospective cohort	MOAKS	46 (65%)	26 ± 5	1 year	30%
Macri et al. [35]	2018	Cross-sectional	Osteophyte equivalent to Kellgren–Lawrence ≥ 2 on plain radiographs	111 (64%)	30 ± 8	1 year	17%
Li et al. [30]	2024	Retrospective cohort	MOAKS	177 (97%)	26 ± 6	2 years	38%
Lee et al. [36]	2018	Retrospective cohort	Grade 2 lesion on second-look arthroscopy, Outerbridge criteria	92 (92%)	30 ± 9	2 years	21%
Patterson et al. [11]	2018	Case–control	Worsening PF cartilage defects, MOAKS	78 (62%)	32 *	1–5 years	44%
Harkey et al. [12]	2022	Cross-sectional	Luyten original criteria	306 (47%)	20 ± 5	6 months	54%
Harkey et al. [16]	2024	Prospective cohort	Luyten original criteria	82 (32%)	19 ± 5	6–12 months	22%
Harkey et al. [10]	2025	Retrospective cohort	Updated Luyten criteria	3693 (48%)	27 ± 7	6 months, 1 year, 2 years	68%, 54%, 46%

* Median age. ACLR, anterior cruciate ligament reconstruction; MOAKS, MRI Osteoarthritis Knee Score; PF, patellofemoral; PFOA, patellofemoral osteoarthritis.

## Data Availability

No new data were created or analyzed in this study. Data sharing is not applicable to this article.

## Supplementary Materials

The following supporting information can be downloaded at: https://www.mdpi.com/article/10.3390/healthcare14142081/s1., Figure S1: Cochrane ROBINS-I V2 for studies assessing risk factors of early PFOA following ACLR; Table S1: Study characteristics and results of studies assessing risk factors of early PFOA following ACLR.

## Abbreviations

The following abbreviations are used in this manuscript:

ACL	Anterior cruciate ligament
ACLR	Anterior cruciate ligament reconstruction
ACR	American College of Rheumatology
AAOS	American Academy of Orthopaedic Surgeons
BLOKS	Boston Leeds Osteoarthritis Knee Score
BMI	Body Mass Index
BPTB	Bone–patellar tendon–bone
ESM	Electronic Supplementary Material
HT	Hamstring tendon
KOOS	Knee injury and Osteoarthritis Outcome Score
MeSH	Medical Subject Headings
MOAKS	MRI Osteoarthritis Knee Score
MOON	Multicenter Orthopaedic Outcomes Network
MRI	Magnetic resonance imaging
OA	Osteoarthritis
OAAA	OA Action Alliance
OARSI	Osteoarthritis Research Society International
PF	Patellofemoral
PFOA	Patellofemoral osteoarthritis
PRP	Platelet-rich plasma
QT	Quadriceps tendon
RCT	Randomized controlled trial
ROBINS-I V2	Risk Of Bias In Non-randomized Studies of Interventions I version 2
SOAR	Stop OsteoARthritis
SUPER	SUpervised exercise-therapy and Patient Education Rehabilitation
WORMS	Whole-Organ Magnetic Resonance Imaging Score
