# Supplementary material for: Early Patellofemoral Osteoarthritis Following ACL Reconstruction: A Narrative Review
Source: Healthcare (Basel). 2026 Jul 12;14(14):2081. doi: 10.3390/healthcare14142081 (PMC13409903; doi:10.3390/healthcare14142081)
Supplement: Supplementary file 1 [file healthcare-14-02081-s001.zip › Supplementary Material Figure S1.pdf]

Electronic Supplementary Material Figure S1: Cochrane risk of bias in non-randomized studies of interventions (ROBINS-I V2) for studies assessing risk factors of early PFOA following ACLR

Article title: *Early Patellofemoral Osteoarthritis following ACL Reconstruction: A Narrative Review*

Journal name: Healthcare

Author names: Carolina Kekki, Christoffer von Essen, Eric Hamrin Senorski, Camilo Helito, Marko Ostojic, Riccardo Cristiani

Corresponding author: Carolina Kekki [1, 2]

Email: Carolina.kekki00@gmail.com

Affiliations:

1 Stockholm Sports Trauma Research Center, Department of Molecular Medicine and Surgery, Karolinska Institutet, 17177 Stockholm, Sweden

2 Capio Artro Clinic, FIFA Medical Centre of Excellence, 11427 Stockholm, Sweden

Supplementary Material Figure S1:

Cochrane ROBINS-I V2 for studies assessing risk factors of early PFOA following ACLR

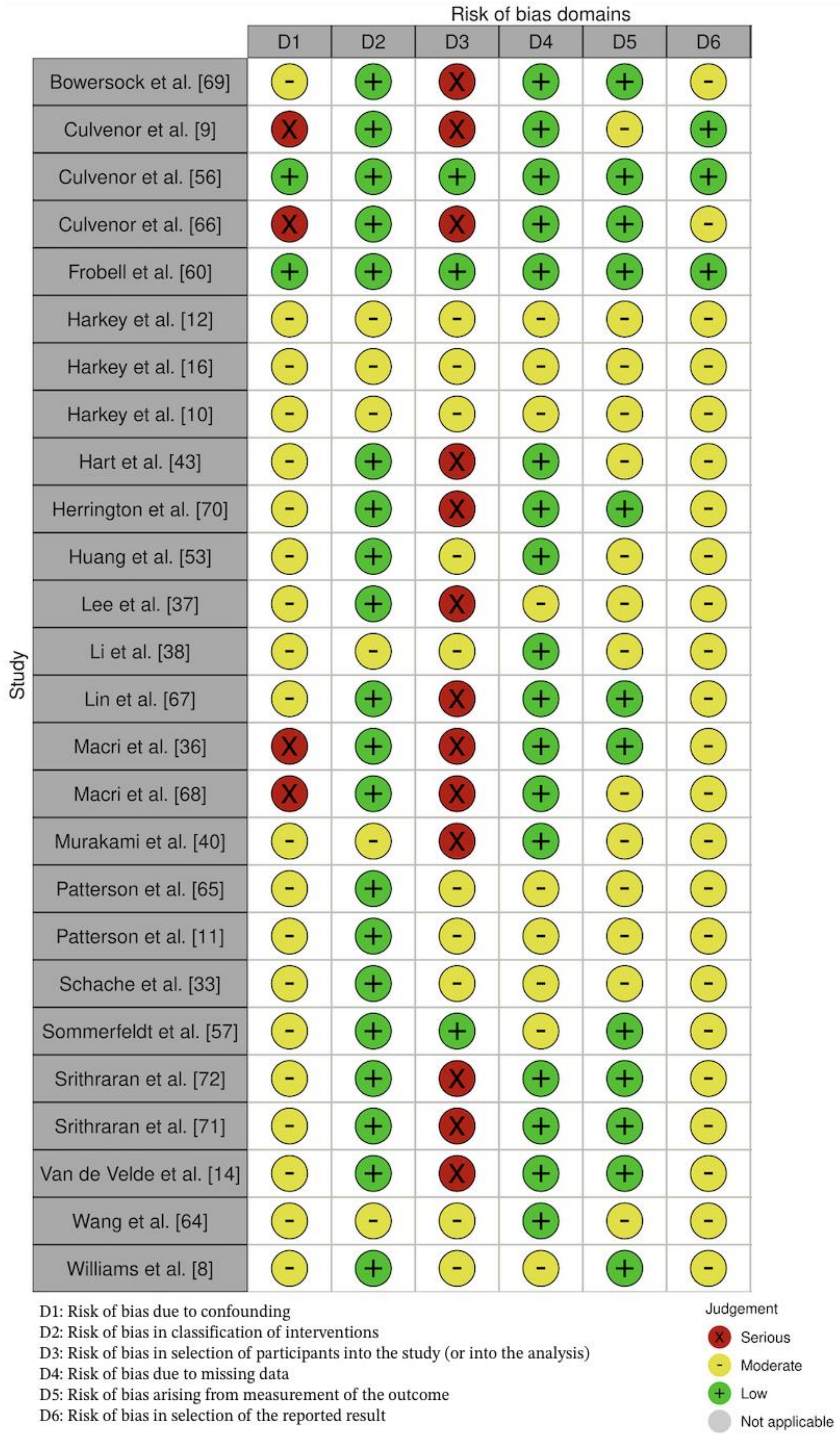

D1; domain 1, D2; domain 2, D3; domain 3, D4; domain 4, D5; domain 5, D6; domain 6, ROBINS-I V2; risk

of bias in non-randomized studies of interventions
