# Supplementary material for: Early Patellofemoral Osteoarthritis Following ACL Reconstruction: A Narrative Review
Source: Healthcare (Basel). 2026 Jul 12;14(14):2081. doi: 10.3390/healthcare14142081 (PMC13409903; doi:10.3390/healthcare14142081)
Supplement: Supplementary file 1 [file healthcare-14-02081-s001.zip › Supplementary Material Table S1.pdf]

Electronic Supplementary Material Table S1: Summary of studies assessing risk factors for early PFOA following ACLR

Article title: *Early Patellofemoral Osteoarthritis following ACL Reconstruction: A Narrative Review*

Journal name: Healthcare

Author names: Carolina Kekki, Christoffer von Essen, Eric Hamrin Senorski, Camilo Helito, Marko Ostojic, Riccardo Cristiani

Corresponding author: Carolina Kekki [1, 2]

Email: Carolina.kekki00@gmail.com

Affiliations:

1 Stockholm Sports Trauma Research Center, Department of Molecular Medicine and Surgery, Karolinska Institutet, 17177 Stockholm, Sweden

2 Capio Artro Clinic, FIFA Medical Centre of Excellence, 11427 Stockholm, Sweden

Supplementary Material Table S1:

Study characteristics and results of studies assessing risk factors of early PFOA following ACLR

Study	Year	Study design	Risk factor	Diagnostic criteria PFOA	Number of patients (%male)	Mean age (years)	Follow-up	Results
Culvenor et al.[9]	2015	Cross-sectional	Age ≥26 years	MOAKS	111 (64%)	30 ± 8.0	1 year	Cartilage lesions: OR: 4.0 (95% CI 1.6-10.3), p<0.05
Lee et al.[36]	2018	Retrospective cohort	Age	Second look arthroscopy	92 (92%)	29.5 ± 9.4	2 years	OR: 1.1 (95% CI 1.0-1.2), p=0.018
Patterson et al.[11]	2018	Case-control	Age >26 years	Worsening PF cartilage defects on MOAKS	78 (62%)	32*	1-5 years	OR: 4.19 (95% CI, 1.78–9.86), p<0.05
Culvenor et al.[9]	2015	Cross-sectional	Sex	MOAKS	111 (64%)	30 ± 8.0	1 year	OA: OR: 4.9 (95% CI 1.0–23.7), p<0.05. Osteophytes: OR: 6.3 (95% CI 2.4–16.2), p<0.05
Lee et al.[36]	2018	Retrospective cohort	Sex	Second look arthroscopy	92 (92%)	29.5 ± 9.4	2 years	OR: 3.2 (95% CI 0.66-15.9), p=0.152
Schache et al.[32]	2023	Prospective cohort	Sex	MOAKS	46 (65%)	26 ± 5	1 and 5 years	79% (11/14) participants with early PFOA at 1 year postoperatively were male
Murakami et al.[38]	2023	Retrospective cohort	Sex	Second look arthroscopy	114 (65%)	27.0 ± 10.1	18 months	Estimate: 0.416 (CI 0.07-0.76), p=0.019
Li et al.[30]	2024	Retrospective cohort	Sex	MOAKS	177 (97%)	26.4 ± 5.6	2 years	OR: 3.07 (95% CI 0.84-11.23), p=0.09
Culvenor et al.[9]	2015	Cross-sectional	BMI >25 kg/m^2	MOAKS	111 (64%)	30 ± 8.0	1 year	Bone marrow lesions: OR: 3.0 (95% CI1.3–6.9), p<0.05

Lee et al.[36]	2018	Retrospective cohort	BMI	Second look arthroscopy	92 (92%)	29.5 ± 9.4	2 years	OR: 0.96 (0.78-1.18), p=0.69
Li et al.[30]	2024	Retrospective cohort	BMI	MOAKS	107 (65%)	29 ± 7	1 year	OR: 0.48 (1.06-0.90), p=1.26
Hart et al.[41]	2024	Cross-sectional	BMI, waist circumference, knee subcutaneous tissue thickness	MOAKS	107 (65%)	29 ± 7	1 year	BMI: OR 1.05 (95% CI 1.00-1.12)<br>Waist circumference: OR 1.02 (95% CI 1.00-1.40)<br>Knee subcutaneous adipose tissue thickness (mm): OR 1.05 (95% CI 1.00-1.09)
Lee et al.[36]	2018	Retrospective cohort	Meniscectomy at primary ACLR	Second look arthroscopy	92 (92%)	29.5 ± 9.4	2 years	OR: 0.09 (95% CI 1.01-1.18), p = 0.02
Murakami et al.[38]	2023	Retrospective cohort	Concomitant meniscal injury/treatment	Second look arthroscopy	114 (65%)	27.0 ± 10.1	18 months	Correlation to second look arthroscopy:<br>Medial meniscus tear/suture/resection: n.s.<br>Lateral meniscus tear/suture/resection: n.s.
Li et al.[30]	2024	Retrospective cohort	Concomitant meniscal injury	MOAKS	107 (65%)	29 ± 7	1 year	OR: 3.18 (95% CI 1.17-8.60), p=0.02
Huang et al.[51]	2022	Retrospective cohort	Concomitant meniscal injury	MRI, Modified Outerbridge criteria	129 (64%)	27.0 ± 6.1	1 year	Lateral meniscal resection: OR: 6.9 (95% CI 1.8-26.3), p=0.004<br>Medial meniscal resection: OR: 6.0 (95% CI 6.0-20.6), p=0.004
Culvenor et al.[9]	2015	Cross-sectional	Time to ACLR <3 months	MOAKS	111 (64%)	30 ± 8.0	1 year	OA: OR: 0.6 (95% CI 0.2–1.8), p=n.s.<br>Bone marrow lesion: OR: 0.9 (95% CI 0.3–2.5), p=n.s.

								Cartilage lesion: OR: 1.1 (95% CI 0.4–2.5), p=n.s. Osteophytes: OR: 1.5 (95% CI 0.6–3.7), p=n.s.
Li et al.[30]	2024	Retrospective cohort	Time to ACLR	MOAKS	107 (65%)	29 ± 7	1 year	OR: 1.04 (95% CI 1.00-1.07), p=0.03
Culvenor et al.[54]	2019	RCT: Early ACLR (within 10 weeks) vs optional delayed ACLR (within 5 years)	Time to ACLR	MRI, PF cartilage thickness	121 (74%)	Early ACLR: 26.6 ± 5.1 Optional delayed ACLR: 25.7 ± 4.7	2 and 5 years	Early ACLR vs optional delayed ACLR group at 2 (−25 µm (−52, 1 µm) +14 µm (−6 to 34 µm), p=0.02) and 5 years (−36 µm (−78 to 5 µm) vs +18 µm (−7, 42 µm), p=0.02
Sommerfeldt et al.[55]	2018	Cross-sectional	Time to ACLR	IKDC grade “mild” or higher	860 (53%)	27.0*(range 12-63)	2 years	n.s. (OR, CI, p not presented)
Murakami et al.[38]	2023	Retrospective cohort	Graft choice: HT vs BPTB	Second look arthroscopy	114 (65%)	27.0 ± 10.1	18 months	Graft choice: n.s.
Frobell et al.[58]	2013	RCT	Graft choice: HT vs BPTB	Sum of the two marginal osteophyte grades from the same compartment 2 or above on plain radiographs	121 (74%)	Early ACLR: 26.4 ± 5.1 Optional delayed ACLR: 25.8 ± 4.7	5 years	Statistically more PFOA (p=0.001) in knees reconstructed with BPTB
Lee et al.[36]	2018	Retrospective cohort	Quadriceps weakness: Peak torque	Second look arthroscopy	92 (92%)	29.5 ± 9.4	2 years	OR: 2.19 (95% CI 1.08-12.44), p=0.031

Murakami et al.[38]	2023	Retrospective cohort	Quadriceps weakness: LSI	Second look arthroscopy	114 (65%)	27.0 ± 10.1	18 months	LSI: 84.2 ± 16.2, p<0.01
Li et al.[30]	2024	Retrospective cohort	Quadriceps weakness: thigh circumference	MOAKS	107 (65%)	29 ± 7	1 year	OR: 0.74, (95% CI 0.599 0.925), p=0.008
Patterson et al.[63]	2020	Prospective cohort	Quadriceps weakness: LSI <90%	MOAKS	78 (62%)	28 ± 15	5 years	OR: 3.66 times (95% CI 1.12-12.01)
Wang et al.[62]	2015	Retrospective cohort	Quadriceps weakness: Peak torque <80% vs contralateral limb	Second look arthroscopy	88 (76%)	27.3 (range 16-42)	2 years	Ipsilateral vs contralateral: 26% vs 48%, p<0.05
Huang et al.[51]	2022	Retrospective cohort	Quadriceps weakness: Peak torque <80% vs contralateral limb	MRI, Modified Outerbridge criteria	129 (64%)	27.0 ± 6.1	1 year	OR: 4.7 (95% CI 1.9-12.1), p=.001
Schache et al.[32]	2023	Prospective cohort	Biomechanics: Peak PF joint contact force	Motion capture camera during standardized forward hop	46 (65%)	26 ± 5	1 and 5 years	Peak PF joint contact force association to PFOA at 1 year postoperatively: PR: 1.37 (95% CI 1.02-1.85), p=0.04<br>Peak PF joint contact force association to PFOA at 5 year postoperatively: RR 1.55 (95% CI 1.13-2.11), p=0.006
Van de Velde et al.[14]	2008	Controlled laboratory	Biomechanics: Patellar rotation, lateral shift in cartilage contact,	MRI-based computer models, Dual-Orthogonal Fluoroscopic Imaging	8 (75%)	Range: 19-38	6 months	Patellar rotation: 2° increased, p<0.05<br>Lateral shift in cartilage contact: 6.5 + 2.2mm to 8.1 + 1.9mm at 15-30°, p<0.05

			patellar tilt vs contralateral limb					Patellar tilt: 7.0° + 3.5° at 0° knee flexion, $p<0.05$
Culvenor et al.[64]	2016	Cross-sectional	Biomechanics: peak knee flexion angles and moments, knee internal rotation excursion during standardized forward hopping task	MOAKS, motion capture camera	45 (67%)	26 ± 5	1-2 years	Peak knee flexion angles: −5.2° (95% CI (-9.9)-(-0.4)), $p=0.04$<br>Peak knee flexion moments: −4.2 Nm/kg.m (95% CI (-7.8)-(-0.6)), $p=0.024$<br>Knee internal rotation excursion: 5.3° (95% CI 2.0-8.6), $p=0.002$
Lin et al.[65]	2019	Prospective cohort	Biomechanics: patellar flexion angle, patellar external rotation, lateral tilt, lateral translation during knee flexion, ACLR vs contralateral limb	CT computer models, Single-plane fluoroscopic dynamic imaging	20 (50%)	Range: 30-37	6 months	Patellar flexion angle: Reduced from 0-60°: $p<0.05$<br>Patellar external rotation: 3-5° increased at 0-60°, $p<0.05$)<br>Lateral tilt: Increased from 0-90°: $p<0.05$<br>Lateral translation: 3mm increased at 0-30°: $p<0.05$
Macri et al.[66]	2019	Longitudinal observational	Biomechanics: Lateral patellar tilt, lateral displacement, trochlear angle	MOAKS, 3D PD VISTA	73 (60%)	29 ± 9	5 years	Lateral patella displacement: OR: 1.09 (95% CI 1.01-1.16), no p-value reported<br>Lateral tilt: OR: 0.91 (95% CI 0.83-0.99), no p-value reported<br>Trochlear angle: OR: 0.88 (95% CI 0.77-1.00), no p-value reported

Bowersock et al.[67]	2017	Experimental laboratory	Biomechanics: PF joint peak force. PF joint loading, ACLR vs contralateral limb	Motion capture camera during running with step lengths: preferred, -5%, -10%	18 (50%)	22 ± 5	4.5 years	PF joint peak force: Reduced in -5%, p<0.05 and -10% step length, p<0.05<br>PF joint loading: Reduced at -10% step length, p<0.05
Herrington et al.[68]	2017	Controlled laboratory	Biomechanics: Knee extensor moment, knee flexion angles, PF contact force, PF contact pressure. ACLR vs contralateral limb	Motion capture camera during running	34 (71%)	21.8 ± 3.9	2 years	Knee extensor moment: p<0.002, p<0.0003<br>Knee flexion angles: p=0.003, p=0.003<br>PF contact force: p=0.03, p=0.04<br>PF contact pressure: p=0.01, 0=0.04
Sritharan et al.[69]	2020	Controlled laboratory	Biomechanics: Peak knee flexion angle, knee extension moment, and PF joint contact force. ACLR vs contralateral limb	Motion capture camera during running	55 (60%)	28 ± 7	1-2 years	Peak knee flexion angle: 46.5 ± 5.3 vs 47.5 ± 5.0, p=0.12<br>Knee extension moment: 14.0 ± 2.4 vs 15.5 ± 2.5, p<0.001<br>PF joint contact force: 6.1 ± 1.3 vs 6.7 ± 1.4, p<0.001
Sritharan et al.[70]	2022	Controlled laboratory	Biomechanics: Peak knee flexion angle, knee extension moment, PF joint	Motion capture camera during single-leg forward hop	66 (64%)	28 ± 6	1-2 years	Peak knee flexion angle: Mean difference: -6° (95% CI (-10°)- (-2°)), p=0.002<br>Knee extension moment: -3.63 (95% CI (-5.29)-(-1.97)), p<0.001

			contact force. ACLR vs contralateral limb					PF joint contact force: -2.24 (95% CI (-3.31)-(-1.18)), p<0.001
Williams et al.[8]	2022	Longitudinal controlled laboratory	Biomechanics: PF contact forces. ACLR vs contralateral limb	EMG during level walking	27 (52%)	23 ± 6	3, 6, 24 months	3 months: Underloading 7-30% of stance, p<0.001<br>6 months: Underloading 11-23%, p = 0.001 and 27-32% of stance, p = 0.025<br>24 months: N.s.
Macri et al.[35]	2018	Cross-sectional	Biomechanics: Patellar alignment, sulcus angle, trochlear angle	Osteophytes on radiograph, equivalent to Kellgren-Lawrence grade ≥2, MRI: Patellar alignment, trochlear morphology	111 (63%)	30 ± 8.5	1 year	Patellar alignment: OR: 1.1 (95% CI 1.0-1.2), p=0.02<br>Sulcus angle: OR: 1.1 (95% CI 1.0-1.2), p=0.02<br>Trochlear angle: OR: 1.2 (95% CI 1.0-1.5), p=0.05

*Median age. ACLR; anterior cruciate ligament reconstruction, BMI; body mass index, BPTB; bone-patellar tendon-bone, CI; confidence interval, CT; computed tomography, EMG; electromyography, HT; hamstrings tendon, IKDC; international knee documentation committee, LSI; limb symmetry index, MOAKS; MRI osteoarthritis knee score, MRI; magnetic resonance imaging, OA; osteoarthritis, OR; odds ratio, p; p-value, PF; patellofemoral, PFOA; patellofemoral osteoarthritis, PR; prevalence ratio, RR; risk ratio, 3D PD VISTA; 3-dimensional proton density volume isotropic turbo spin echo acquisition.
